# Much More than Nutrients: The Protective Effects of Nutraceuticals on the Blood–Brain Barrier in Diseases

**DOI:** 10.3390/nu17050766

**Published:** 2025-02-21

**Authors:** Anna E. Kocsis, Nóra Kucsápszky, Ana Raquel Santa-Maria, Attila Hunyadi, Mária A. Deli, Fruzsina R. Walter

**Affiliations:** 1 Biological Barriers Research Group, Institute of Biophysics, HUN-REN Biological Research Centre, H-6726 Szeged, Hungary; annakocsis.work@gmail.com (A.E.K.); kucsapszky.nora@brc.hu (N.K.); 2 Wyss Institute for Biologically Inspired Engineering, Harvard University, Boston, MA 02115, USA; 3 Institute of Pharmacognosy, University of Szeged, Eötvös u. 6, H-6720 Szeged, Hungary; hunyadi.attila@szte.hu; 4 Interdisciplinary Centre of Natural Products, University of Szeged, Eötvös u. 6, H-6720 Szeged, Hungary; 5 HUN-REN-SZTE Biologically Active Natural Products Research Group, Eötvös u. 6, H-6720 Szeged, Hungary; 6 Graduate Institute of Natural Products, Kaohsiung Medical University, Shih-Chuan 1st Rd. 100, Kaohsiung 807, Taiwan

**Keywords:** blood–brain barrier, brain endothelial cell, central nervous system disease, efflux transporter, nutraceutical, plant-derived compound, protection, signal transduction, solute carrier

## Abstract

The dysfunction of the blood–brain barrier (BBB) is well described in several diseases, and is considered a pathological factor in many neurological disorders. This review summarizes the most important groups of natural compounds, including alkaloids, flavonoids, anthocyanidines, carotenoids, lipids, and vitamins that were investigated for their potential protective effects on brain endothelium. The brain penetration of these compounds and their interaction with BBB efflux transporters and solute carriers are discussed. The cerebrovascular endothelium is considered a therapeutic target for natural compounds in diseases. In preclinical studies modeling systemic and central nervous system diseases, nutraceuticals exerted beneficial effects on the BBB. In vivo, they decreased BBB permeability, brain edema, astrocyte swelling, and morphological changes in the vessel structure and basal lamina. At the level of brain endothelial cells, nutraceuticals increased cell survival and decreased apoptosis. From the general endothelial functions, decreased angiogenesis and increased levels of vasodilating agents were demonstrated. From the BBB functions, elevated barrier integrity by tightened intercellular junctions, and increased expression and activity of BBB transporters, such as efflux pumps, solute carriers, and metabolic enzymes, were shown. Nutraceuticals enhanced the antioxidative defense and exerted anti-inflammatory effects at the BBB. The most important signaling changes mediating the increased cell survival and BBB stability were the activation of the WNT, PI3K-AKT, and NRF2 pathways, and inhibition of the MAPK, JNK, ERK, and NF-κB pathways. Nutraceuticals represent a valuable source of new potentially therapeutic molecules to treat brain diseases by protecting the BBB.

## 1. Importance of Nutraceuticals in Health and Disease

The study of nutraceuticals has gained significant attention in recent years, as they offer a promising approach to maintaining and promoting human health. The term nutraceutical is a hybrid expression between nutrition and pharmaceutical, referring to any bioactive compound that can provide both nutritional and medicinal value. The vast majority of such compounds in the human diet are of herbal origin, i.e., they are bioactive constituents of edible plants. These herbal compounds have benefits in a wide range of therapeutic areas in many formulations [1,2]. Numerous studies have highlighted the potential of nutraceuticals in the prevention and management of chronic diseases, such as cardiovascular diseases, cancer, osteoarthritis, diabetes, and neurological disorders [3,4,5,6,7,8,9,10].

The blood–brain barrier (BBB) is one of the major interfaces between the systemic circulation and the brain sustaining fundamental homeostasis for brain functions. For detailed reviews about the main cell types, transporters, basal membranes, glycocalyx, and signal transduction, see [11,12,13,14,15]. Brain endothelial cells comprising the functional basis for the BBB are one of the main cellular targets for dietary natural products [16]. Here, we focus on factors of the special relationship between nutraceuticals and the BBB. BBB penetration of nutraceuticals, and their interaction with influx and efflux transporters and intracellular signaling pathways, will be discussed. The effect of nutraceuticals on BBB protection based on in vitro and in vivo studies draws attention to the versatile use of these mostly plant-derived natural products.

## 2. Major Properties of Nutraceuticals with Protective Effects on the Blood–Brain Barrier

A wide range of natural products have been reported for their BBB protective activity, and these cover all major classes of secondary plant metabolites, including terpenoids, phenolic compounds, alkaloids, and organosulfur compounds, as well as some primary metabolites such as ⍵-3 fatty acids and several vitamins (Table 1). In line with this chemical diversity, it is not surprising that key BBB-related physicochemical characteristics of these compounds represent a very large variation. Molecular masses vary between 148 (cinnamic acid) and 659 (fucoxanthin), logarithm of the octanol-water partition coefficient (logP) values vary between −1.6 (ascorbic acid) and 15.6 (lycopene), and topological polar surface area (TPSA) values vary between 0 for lycopene and β-carotene, and 266 for rutin (see Supplementary Materials, Table S1). These ranges are far wider than what would generally be considered ideal for a central nervous system (CNS) active drug candidate [17]. While there are some highly polar compounds that reportedly protect the BBB, like the cationic anthocyanidines and their glucosides, most of the compounds are rather lipophilic up to the extreme, e.g., the tetraterpene carotenoids. While both extremes in terms of hydrophilic–lipophilic properties are well known for manifesting in problems with biodistribution and making it difficult for a compound to pass through the BBB, it is worth stressing that BBB protecting agents do not necessarily need to penetrate through the barrier to exert their effect [14]. Nonetheless, carotenoids provide an interesting example for a highly lipophilic group of nutraceuticals whose members do not become stuck in extracellular membranes, even without an influx mechanism assisting their uptake. Due to their length matching the thickness of phospholipid bilayers, they are perfectly capable of passing through the BBB by passive diffusion [18].

As seen from Table 1, all nutraceuticals identified as BBB-protective hits had been linked to antioxidant activities. The vast majority of them are well-known ‘classical’ antioxidants, such as phenolic compounds able to scavenge toxic free radicals and mitigate oxidative stress-related cellular damage. This is not surprising, considering the key role of oxidative stress in the onset and progression of BBB damage in diseases [14].

**Table 1:** Blood–brain barrier protective nutraceuticals and natural compounds.

Nutraceutical	Properties	Source	Antioxidant	Efflux Pump Interaction Y/N	Influx Transport Interaction	BBB/Brain Penetration
Alkaloids						
Caffeine	194 Da A	Coffee, green and black tea, guarana berries	Yes [19]	No [20]	CNT2/SLC28A2 [21] OAT1/SLC22A6 [22] GLUT1/SLC2A1 [23]	Yes, high [24,25]
Capsaicin	305 Da A	Chili pepper	Yes [26]	Yes [27,28]	ND	Yes, high [29]
Theophylline	180 Da A	Cocoa beans, brewed tea	Yes [30]	No [31]	OAT1 [22]	Yes [32]
Anthocyanidines						
Cyanidin/Cyanidin-3-O-beta-glucoside/Procyanidine	287 Da H	Red wine, elderflower, berries, tea, apple, cinnamon	Yes * [33]	Yes [34,35]	GLUT1/SLC2A1 [36]	Yes [36,37]
Malvidin/Malvidin-3-O-glucoside	331 Da H	Red wine, berries	Yes [33,38]	Yes [34]	GLUT1/SLC2A1, GLUT3/SLC2A3 [39]	Yes [40]
Carotenoids						
Astaxanthin	597 Da L	Seafood, salmon, trout, algae	Yes *	Yes [41]	ND	Yes *
β-Carotene	537 Da L	Carrots, sweet potato, pumpkin	Yes *	Yes [31,42]	GLUT4/SLC2A4 [43]	No *
Fucoxanthin	659 Da L	Brown algae	Yes * [44]	Yes [45]	SLC7A11 [44]	ND *
Lutein	569 Da L	Kale, spinach, orange, egg yolk, avocado	Yes *	Yes [46]	SR-B1 [47]	Yes *
Lycopene	537 Da L	Tomato, watermelon grapefruit, pomegranate	Yes *	Yes [48]	ND	Yes *
Diarylheptanoids						
Curcumin	368 Da L	Turmeric	Yes * [49]	Yes [50,51]	GLUT1/SLC2A1 [52]	No [53]
Flavonoids						
Apigenin	270 Da L	Parsley, celery, chamomile tea	Yes [54,55]	Yes [56,57]	GLUT1/SLC2A1 [58]	Yes, low [56,59]
Catechin/ Epicatechin	290 Da L	Tea, red wine, cocoa	Yes [60,61]	Yes [62,63]	ASBT/SLC10A2 [64]	Yes [36]
Chrysin	254 Da L	Chamomile, honey, propolis, passion fruit	Yes [65,66]	Yes [67,68]	OATP [69]	ND
Daidzein	254 Da L	Soy, soy products	Yes [70]	Yes [71,72]	GLUT4/SLC2A4 [73]	Yes, high [59]
Fisetin	286 Da L	Onions, leeks, broccoli	Yes [74]	Yes [75]	GLUT4/SLC2A4 [76]	Yes, low [59]
Genistein	270 Da A	Soy, soy products	Yes * [77]	Yes [72,78]	Noradrenalin, serotonin transporter [79] GLUT1/SLC2A1 [80]	Yes, high [56,59]
Hesperetin	302 Da L	Citrus fruit, herbs, wine	Yes * [81,82]	Yes [67,83]	ND	Yes [37]
Hesperidin	611 Da H	Citrus fruit, herbs, wine	Yes [84,85]	Yes [68,86]	ND	Yes, low [56]
Kaempferol	286 Da L	Onion, leeks, broccoli, ginkgo biloba	Yes [87,88]	Yes [67,71]	GLUT4/SLC2A4 [89]	Yes, low [56,90]
Luteolin	286 Da L	Pepper, leafy greens, celery, broccoli	Yes [91]	Yes [92]	SLC7A11 [93]	Yes, low [59]
Myricetin	318 Da L	Onions, leeks, broccoli	Yes [87]	Yes [94]	PCFT/SLC46A1 [95]	Yes [90]
Naringenin	272 Da L	Citrus fruits, herbs, wine	Yes [96]	Yes [65]	ND	Yes [35]
Naringin	581 Da H	Citrus fruits, herbs, wine	Yes [84]	Yes [68,97]	ND	ND
Quercetin	302 Da L	Onion, broccoli, ginkgo biloba, apple	Yes *	Yes [67,98]	SLC7A11 [99]	Yes, low [40,56,90]
Rutin	611 Da H	Citrus fruits, herbs, wine	Yes *	Yes [56,100]	OATP2B1/SLCO2B1 [101] GLUT4/SLC2A4 [102]	Yes, low [56,59]
Silybin/ Silymarin	482 Da L	Milk thistle	Yes * [103]	Yes [68]	ND	ND
Tangeretin	372 Da L	Tangerine, citrus peel	Yes [104,105]	Yes [106]	SGLT1/SLC5A1 [107]	Yes [108]
Monoterpenes						
Borneol	154 Da L	Coriander, ginger oil, rosemary, thyme	Yes [109]	Yes [110,111]	ND	Yes [112]
Carvacrol	150 Da L	Oregano, thyme	Yes [113]	Yes [114]	ND	ND
Omega-3 fatty acids						
Docosahexaenoic acid	328 Da L	Oceanic fish oil, seaweed	Yes [115]	Yes [116]	MFSD2A [117]	Yes [117]
Eicosapentaenoic acid	302 Da L	Fish oil, seaweed	Yes [118,119]	Yes [116]	VNUT/LC17A9 [120]	Yes [121]
Organosulfur compounds						
α-Lipoic acid	206 Da L	Broccoli, yeast, meat, kidney, heart, liver	Yes [122,123]	Yes [124]	SMVT/SLC5A6 [125]	Yes [126]
Sulforaphane	177 Da L	Broccoli, kale, cauliflower	Yes *	Yes [127]	ND	Yes [128]
Phenolic acids						
Caffeic acid	180 Da L	Berries, kiwi, plum, apple	Yes [129]	Yes [130]	MCT1/SLC16A1, MCT4/SLC16A3 [131]	Yes [132]
Cinnamic acid	148 Da L	Cinnamon, grape, cocoa	Yes [133]	Yes [134]	MCT1/SLC16A1, MCT4/SLC16A3 [131]	ND
*p*-Coumaric acid	164 Da L	Berries, kiwi, plum, apple	Yes [135]	ND	MCT1/SLC16A1, MCT4/SLC16A3 [131] OAT3/SLC22A8 [136]	ND
Ferulic acid	194 Da L	Grains, nuts, fruits, vegetables	Yes [137,138]	Yes [139]	MCT1/SLC16A1, MCT4/SLC16A3 [130,140] OAT3/SLC22A8 [136]	Yes, low [25]
Gallic acid	170 Da L	Berries, kiwi, plum, apple	Yes [141]	Yes [134]	OAT3/SLC22A8 [136]	Yes [142]
Rosmarinic acid	360 Da L	Berries, kiwi, plum, apple	Yes [143,144,145]	Yes [146]	OAT1/SLC22A6, OAT3/SLC22A8 [147]	Yes, low [132]
Stilbenes						
Piceatannol	244 Da L	Grape, white tea, passion fruit	Yes [148]	No [149]	MCT1/SLC16A1, MCT4/SLC16A3 [150]	ND
Polydatin	390 Da L	Grapes, cocoa, peanuts	Yes [151]	Yes [152]	ND	Yes, low [153]
Pterostilbene	256 Da L	Blueberries, grapes	Yes [154]	ND	MCT1/SLC16A1, MCT4/SLC16A3 [150]	Yes [155]
Resveratrol	228 Da L	Grapes, wine, peanuts	Yes * [33]	Yes [156]	ND	Yes, low [59,157]
Vitamins						
Vitamin C/ Ascorbic acid	176 Da H	Fruits, vegetables	Yes * [158]	No [159]	SLC19A1, SLC23A2 [11]	Yes [160]
Vitamin B9/ Folic acid	441 Da H	Fruits, vegetables, nuts	Yes [161]	ND	SLC19A1, SLC46A1 [11]	Yes [162]
Vitamin D3/ Cholecalciferol	385 Da L	Fish, milk, meat	Yes [163]	ND	LRPs [11]	Yes [164]
Vitamin E/ α-Tocopherol	431 Da L	Plant oil, nuts, nut oil, spinach, broccoli	Yes *	Yes [165]	SR-B1, αTTP, PLTP [162]	Yes [165]

Abbreviations: A: amphiphilic; ASBT: Ileal apical sodium-dependent bile acid transporter; GLUT: glucose transporter; H: hydrophilic; OAT: organic anion transporter; SMVT: sodium-dependent multivitamin transporter; L: lipophilic; LRP: LDL receptor-related protein; MCT: monocarboxylic acid transporter; MFSD2A: sodium-dependent lysophosphatidylcholine symporter 1; ND: no data; OATP: organic anion-transporting polypeptide; PCFT: proton-coupled folate transporter; PLTP: phospholipid transfer protein; SGLT1: sodium/glucose cotransporter 1; SLC: solute carrier; SR-B1: scavenger receptor class B type 1; αTTP: α-tocopherol (binding) transport protein; *: [10].

This notion has likely been driving related studies and the selection of compounds to be tested as potential BBB protective agents. However, it is now well understood that antioxidant action is much more complex than free radical scavenging, and is based on the modulation of complex signaling pathways to activate endogenous, primarily enzymatic antioxidant defense mechanisms [166]. This is also demonstrated by the appearance of some non-phenolic antioxidants among the BBB protective dietary compounds. Based on the above, we may postulate that there is much more to BBB protection than free radical scavenging by dietary antioxidants, and that a broad chemical space is still waiting to be explored in this regard.

It should also be noted that several of the compounds presented in Table 1 are well known for their low chemical stability and/or bioavailability. This is particularly true for some phenolic compounds, e.g., curcumin and resveratrol. In such cases, the experimental model may fundamentally determine the outcome of a study. Because of this, while BBB protection may have rightfully been connected to the consumption of these compounds, their decomposition products or metabolites may have played a significant role in the effect [166].

Studies focusing on nutraceuticals affecting CNS functions often neglect the amount of the compound that enters the brain, or how the molecule can cross the BBB. Passage of natural compounds across the BBB was reviewed by Ureña-Vacas et al. [167]. We listed 48 compounds in Table 1, and most of them were studied for BBB or brain penetration either in animal or culture models. We did not find permeability data for eight compounds, namely carvacrol, chrysin, cinnamic acid, p-coumaric acid fucoxanthin, naringin, piceatannol, and silymarin (Table 1). Two well-known lipophilic compounds, β-carotene [10] and curcumin [53], are considered to not cross the BBB, which means that their permeability is so low that it is below the detection level. Very low barrier permeability was measured for four molecules, namely fisetin, luteolin, resveratrol, and rutin [59], and from these, only rutin is hydrophilic (Table 1). Low but measurable BBB penetration was described for seven compounds, namely apigenin, ferulic acid, hesperidin, kaempferol, polydatin, quercetin, and rosmarinic acid (Table 1). High permeability was detected for caffeine, which is used as a permeability marker in BBB studies [24], capsaicin when given systematically [29], daidzein, and genistein [59]. For the remaining 23 compounds, BBB crossing was described (Table 1). We found two comparative studies to evaluate the barrier crossing of several herbal compounds: Shimazu et al. tested BBB permeation in rats and in a rat BBB co-culture model [59], while Yang et al. used rat brain endothelial- and intestinal epithelial-based culture models [56]. A clear ranking for penetration can be established only in comparative studies [56,59]. The limitation of most studies is that they investigate only one compound; therefore, the levels of permeability and brain penetration cannot be compared to other datasets due to differences in models, methods, and calculations [167]. Lipophilicity and passive transport are not the only determinants of BBB crossing for the compounds listed in Table 1; stability, bioavailability and, most importantly, brain endothelial transporters can also play a role.

Active transport systems at the level of brain endothelial cells play an important role in the entry of nutrients to the brain [11]. Nutraceuticals and their glucoside derivatives interact with hexose transporters, such as GLUT-1, -2, and -4, which help their transport across biological barriers. Members of the solute carrier family including organic cationic and anionic transporters (OCTs and OATs), sodium-coupled nucleoside transporters (CNTs), organic anion transporters (OATPs), and monocarboxylate transporters (MCTs), are highly expressed at the BBB, as demonstrated by transcriptomic studies on mouse [168,169,170] and human [171,172] isolated brain microvessels, and also on cultured brain endothelial cells [173,174,175,176].

The effect of nutraceuticals and different herbal compounds on efflux pumps is a well-studied area, and has been described in detail [11,177,178,179,180]. As a general conclusion, multiple herbal compounds interact as ligands or inhibitors with major efflux pumps, which are also present at the BBB, such as P-glycoprotein (PGP, MDR1/ABCB1) or breast cancer resistant protein (BCRP/ABCG2). These compounds can interact with the efflux pumps, decreasing their bioavailability. Upon inhibition, other nutrients or drugs otherwise subject to efflux pump activity can reach the target tissue in a different concentrations, leading to higher absorption and changing the dose delivered to the tissue [181].

**Figure 1:**
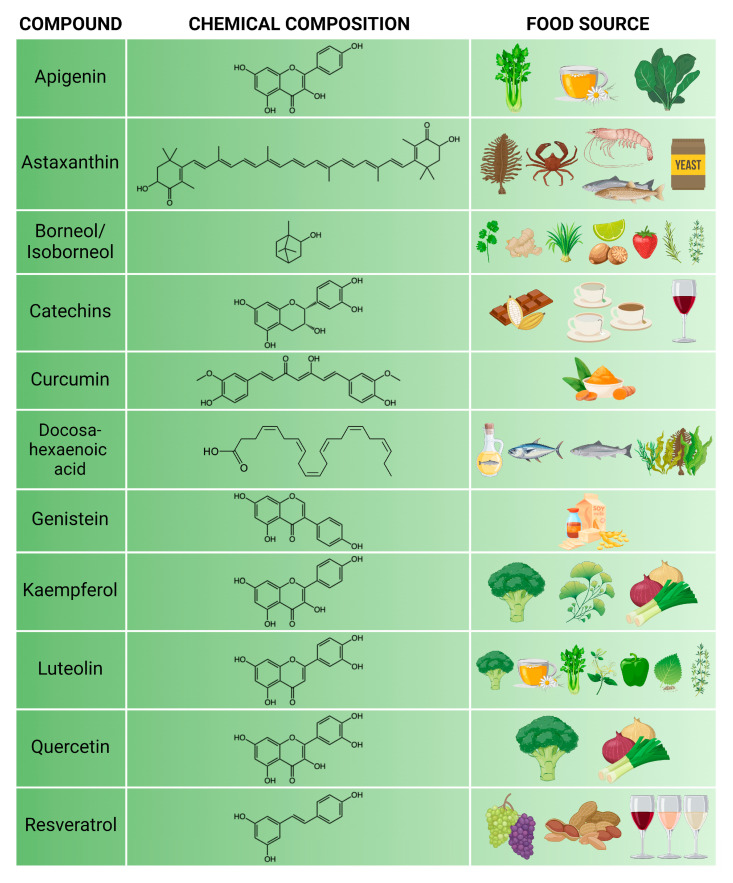
The most tested nutraceuticals for protective effects on the blood–brain barrier both in vivo and in vitro. The chemical composition and some of the food sources of the selected 11 compounds are shown. Created in BioRender. Kucsápszky N., Santa Maria, A. (2025) https://BioRender.com/j91e333 (accessed on 10 February 2025).

From the compounds listed in Table 1, those that were tested for protective effects on both in vivo and culture models of the BBB (Table 2 and Table 3) by more than two research groups are shown in Figure 1. Among the selected 11 compounds, 6 are flavonoids, namely apigenin, catechins, genistein, luteolin, kaempferol and quercetin. There could be several reasons for why flavonoids are the most investigated chemical group for their BBB effects. Firstly, they are present in vegetables such as leafy greens, onions, herbs, and beverages, therefore they are regularly consumed in balanced diets. Secondly, these compounds are also major constituents in many traditional medicines, such as *Gingko biloba* and chamomile tea. Thirdly, they are widely studied for neuroprotection in preclinical models [182], although much less for their effects on the BBB. The main biomedical research field for curcumin, a diarylheptanoid compound in the spice turmeric, and resveratrol, a stilbene found in grapes, wine, and nuts, is cancer [183], although their role is also investigated in cardiovascular and CNS diseases [184]. It was surprising to find only a handful of studies for these two compounds on BBB models. Regarding the carotenoid astaxanthin and the polyunsaturated fatty acid DHA, their primary food source is fish, seafood, and algae (Figure 1), which are well known for their beneficial effects on general health, brain development, and cognition [185]. The monoterpene borneol that can be found mainly in spices and herbs is mainly investigated for its use to help drug delivery across biological barriers [186].

## 3. Protective Effects of Nutraceuticals on the BBB in CNS Diseases

**Figure 2:**
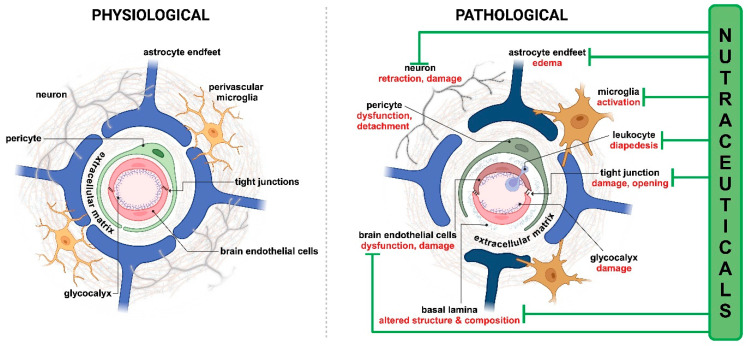
The structure of the blood–brain barrier in physiological and pathological conditions. The schematic drawing shows the protective effects of nutraceuticals on major components of BBB dysfunction. Created in BioRender. Kucsápszky N., Santa Maria, A. (2025) https://BioRender.com/p74u637 (accessed on 10 February 2025).

While the BBB is essential for preserving neural functions, its disruption or dysfunction is implicated in a wide range of CNS pathologies. Conditions such as neurodegenerative diseases (Alzheimer’s disease, Parkinson’s disease), cerebrovascular disorders (subarachnoid hemorrhage, stroke, ischemia), and traumatic brain injury (TBI), along with different disease states such as hyperlipidemia, oxidative stress, or neurodevelopmental conditions like autism, often involve alterations in BBB integrity and function [187]. These changes can lead to an imbalance in transport mechanisms and intercellular junctional molecule expression, elevation of proinflammatory cytokine levels, and disruption of blood flow, all exacerbating disease progression [12]. Nutraceuticals in preclinical studies could partially or fully restore barrier integrity, decrease brain edema, and block the downregulation of junctional molecule expression, along with reducing oxidative stress and inflammation and restoring healthy BBB phenotypes in CNS pathologies, as summarized in Figure 2.

### 3.1. In Vivo Investigations

The use of animal models is fundamental in biomedical research to advance our understanding of the pathophysiology, progression, and treatment of diseases [188]. The protective effects of nutraceuticals on BBB were mainly studied in rat or mouse disease models (Table 2).

**Table 2:** The effects of nutraceuticals on the blood–brain barrier model in animal models of diseases.

Compound	Disease Model	Effects on BBB Parameters	Reference
Apigenin	subarachnoid hemorrhage, rat	inflammation ↓, BBB disruption ↓, ZO-1, occludin ↑	[189]
	cerebral IR, MCAO, rat	vascularization/tube formation ↑, cerebral infarction ↓	[190]
Astaxanthin and derivatives	subarachnoid hemorrhage, mouse	BBB disruption ↓	[191]
	subarachnoid hemorrhage, rat	barrier integrity ↑, brain edema ↓, IL-1β, TNF-α, MMP-9 expression ↓	[192]
Borneol	-	R123 permeability in hippocampus ↑, PGP and MRP1 ↓, TJ disruption	[193]
	cerebral IR -	blood pressure, cerebrovascular resistance ↓, edema ↓, BBB integrity, eNOS, CLDN-5, ZO-1 ↑, ET-1, iNOS, MMP-2/9, ICAM1, LFA-1 ↓	[111,194]
Caffeic acid phenethyl ester	TBI, rat and mouse	vascular integrity ↑, CLDN-5 ↑	[195]
β-carotene	cerebral IR, MCAO, mouse	barrier integrity ↑, occludin, ZO-1 ↑ peroxynitrite generation ↓	[196]
Carvacrol	TBI, rat	barrier integrity ↑, brain edema ↓, occludin, CLDN-5, ZO-1 ↑, MMP-9 ↓	[113]
Catechin	TBI, rat	barrier integrity ↑, ZO-1, occludin ↑	[197]
Chrysin	TBI, rat	barrier integrity ↑, brain EB content ↓	[198]
*p*-Coumaric acid	hypoxia, mouse	barrier integrity ↑, brain edema ↓, occludin expression ↑	[199]
Curcumin	cerebral IR, MCAO, rat	barrier integrity ↑, brain EB content ↓	[200]
	Hypoxia/hypercap-nia, rat	brain edema ↓, apoptosis ↓, AQP4 levels ↓,	[201]
Daidzein	cerebral IR, MCAO, rat	barrier integrity ↑, astrocyte swelling ↓, cytoplasmic vacuolation ↓, edema ↓, vessel lumen ↑	[202]
Docosahexaenoic acid	cerebral IR, MCAO, rat	barrier integrity ↑, brain edema ↓	[203]
	cerebral IR, MCAO, rat	barrier integrity ↑	[204]
	cerebral IR, left CCAO, rat	barrier integrity ↑, brain edema ↓, occludin, CLDN-5, ZO-1 ↑, MMP-2/9 ↓	[205]
	uremia + contrast media, mouse	CLDN-5, laminin α-4, -5 ↑	[206]
Eicosapentaenoic acid	cerebral IR, left CCAO, rat	barrier integrity ↑, brain edema ↓, occludin, CLDN-5, ZO-1 ↑, MMP-2/9 ↓	[205]
	uremia + contrast media, mouse	CLDN-5, laminin α-4, -5 ↑	[206]
Epigallo-catechin gallate	cerebral IR, MCAO, rat	barrier integrity ↑, TJ opening ↓ occludin, CLDN-5, ZO-1 expression ↑	[207]
Ferulic acid + tetramethylpyrazine	cerebral IR, MCAO, rat	barrier integrity ↑, brain edema ↓, JAM-1, occludin ↑, MMP-9 expression ↓	[208]
Fisetin	autism, valproic acid-induced, rat	barrier integrity ↑, CLDN-5 expression ↑	[209]
Fucoxanthin	TBI, mouse	barrier integrity ↑, brain edema ↓, occludin, CLDN-5, ZO-1, VE cadherin ↑, MMP-9 ↓, apoptosis and ferroptosis ↓, BMEC mitophagy ↑	[210]
Genistein	TBI, rat	barrier integrity ↑	[211]
Hesperetin	TBI, mouse	barrier integrity ↑, brain edema ↓, ZO-1, occludin, CLDN-5 ↑, NLRP3 inflammasome ↓	[212]
Hesperidin	cerebral IR, MCAO, mouse	barrier integrity ↑, brain edema ↓, disruption of CLDN-5 and ZO-1 ↓	[85]
Kaempferol	neuroinflammation, LPS-induced, mouse	barrier integrity ↑, occludin, connexin-43 expression ↑	[213]
	neuroinflammation and BBB dysfunction, LPS-induced	BBB structure restored, brain edema ↓, occludin, connexin-43 expression ↑	[214]
Kaempferol-glucoside/Juglanin	cerebral IR, MCAO, mouse	BBB permeability ↓, VEGF and VEGFR2 ↓ ZO-1, occludin expression ↑	[215]
α-Lipoic acid	TBI, rat	barrier integrity ↑, brain EB content ↓	[216]
Lutein	subarachnoid hemorrhage, rat	vasospasm ↓	[217]
	TBI, rat	IL-1, IL-6, TNF-α, CCL2 ↓, ROS ↓, SOD, GSH ↑, ICAM-1, ET-1 ↓	[218]
Luteolin	AD, Aβ_25–35_-induced, mouse	BBB leakage ↓, astrocyte swelling ↓, CBF ↑ ZO-1, occludin, CLDN-5 expression ↑,	[219]
	diabetes, high-fat diet and streptozotocin- induced, rat	ZO-1, occludin and GLUT-1 expression ↑	[220]
Lycopene	subarachnoid hemorrhage, rat	barrier integrity ↑, brain edema ↓	[221]
	hyperlipidemia, high fat diet induced, rat	VEGF, VCAM-1 ↓, CLDN-5 ↑, IL-1, IL-6, and TNF-α ↓	[222]
Malvidin	cerebral IR, BCCAO, rat	eNOS ↑, MMP-9 ↓	[223]
Naringenin	cerebral IR, MCAO, mouse	BBB leakage ↓, ZO-1, occludin, CLDN-5, β-catenin ↑	[224]
Polydatin	cerebral IR, MCAO, rat	barrier integrity ↑, brain edema ↓, CLDN-5 expression ↑	[225]
	cerebral IR, MCAO, rat	barrier integrity ↑, brain edema ↓, ZO-1, occludin, CLDN-5 ↑, TNF-α, IL-1β, IL-6, CCL2 levels ↓, ICAM-1 and VCAM-1 ↓	[226]
Procyanidin B2	cerebral IR, MCAO, rat	barrier integrity ↑, brain edema ↓, ZO-1 expression ↑	[227]
Pterostilbene	cerebral IR, MCAO, rat	barrier integrity ↑, CBF ↑, laminin ↑, ZO-1, occludin, CLDN-5, VE-cadherin ↑	[228]
	cerebral IR, MCAO, rat	barrier integrity ↑, brain edema ↓, MMP-2/9 expression ↓	[229]
Quercetin	AD, Aβ_25–35_-induced, mouse	barrier integrity ↑, CBF ↑	[230]
	cerebral ischemia, photothrombosis-induced, rat	barrier integrity ↑, MMP-9 activity ↓	[231]
	cerebral IR, BCCAO, rat	barrier integrity ↑, brain endothelial cell swelling ↓, vesicles and vacuoles ↓, CLDN-5, ZO-1, β-catenin ↑, MMP-9 ↓	[232]
	oxidative stress, PCB-induced, rat	occludin, CLDN-5, JAM-3, ZO-1, AF-6 ↑	[233]
	cerebral IR, MCAO, rat	barrier integrity ↑, occludin, CLDN-5, ZO-1 expression ↑	[234]
Quercetin +/− hydroxylsafflor yellow A	cerebral IR, MCAO, mouse	barrier integrity ↑	[235]
Resveratrol	recurrent ischemic stroke, rat	barrier integrity ↑, brain edema ↓, no change in CBF	[236]
Rosmarinic acid	MCAO + diabetes, STZ-induced, rat	barrier integrity ↑, brain edema ↓	[237]
Tangeretin	cerebral IR, MCAO, rat	barrier integrity ↑	[238]
Vitamin B9	sepsis, cecal ligation and perforation, rat	barrier integrity ↑	[239]
Vitamin C	cerebral IR, MCAO, rat	barrier integrity ↑, MMP-2/9 expression ↓, CLDN-1,CLDN-5, ZO-1 ↑	[240]
Vitamin D3	TBI, rat	barrier integrity ↑, brain edema ↓, ZO-1, occludin expression ↑	[241]

Abbreviations: AD: Alzheimer’s disease; AF-6: ALL-1 fusion partner at chromosome-6; BCCAO: bilateral common carotid artery occlusion; BMEC: brain microvascular endothelial cell; CBF: cerebral blood flow; CCL2: chemokine (C-C motif) ligand 2; CLDN: claudin; EB: Evans blue; eNOS: endothelial nitric oxide synthase; ET-1: endothelin-1; GLUT-1: glucose transporter 1; ICAM1: intercellular adhesion molecule 1; IL: interleukin; iNOS: inducible nitric oxide synthase; IR: ischemia/reperfusion; JAM: junction associated molecule; LFA-1: lymphocyte function-associated antigen 1; MMP: matrix metalloproteinase; NLRP3: microglial nodd-like receptor protein 3; PCB: polychlorinated biphenyl; ROS: reactive oxygen stress; STZ: streptozotocin; TBI: traumatic brain injury; MCAO: transient middle cerebral artery occlusion; TNF-α: tumor necrosis factor-α; VCAM-1: vascular cell adhesion protein 1; ZO-1: zonula occludens-1; ↑: increase; ↓: decrease.

#### 3.1.1. Traumatic Brain Injury

Mechanical traumas affecting the head or spine are the major causes of TBI, in which BBB dysfunction leads to secondary neuronal injuries. BBB breakdown in TBI includes the loss of tight junctions (TJs), increased vesicular transport, extravasation of plasma proteins, and brain edema, causing further neuronal damage [242,243]. Decreased edema and increased BBB integrity after TBI were found after treatment with α-lipoic acid [216], chrysin [198], genistein [211], carvacrol [113], and vitamin D3 [241]. Caffeic acid phenethyl ester also increased BBB integrity by elevating claudin-5 levels [195]. Catechin and hesperetin acted similarly by restoring zonula occludens protein 1 (ZO-1) and occludin expression [197,212]. Hesperetin reduced the NLRP3 inflammasome activation after TBI [212]. Carvacrol protected BBB properties after trauma due to its antioxidant property, restored the expression of junctional proteins, and decreased the level of matrix metalloproteinase-9 (MMP-9; [113]). Lutein in a rat TBI model decreased the level of intercellular adhesion molecule-1 (ICAM-1) and endothelin-1, a potent vasoconstrictor, and elevated nuclear factor erythroid 2-related factor 2 (Nrf-2) expression and antioxidant cell response [218]. Fucoxanthin, similarly to previous molecules, rescued the barrier integrity, increased the expression of junctional proteins, attenuated brain endothelial cell apoptosis and ferroptosis, and increased mitophagy [210].

#### 3.1.2. Cerebrovascular Disorders

In the aging population, the most common CNS diseases are those that affect the cerebral vasculature. Ischemic stroke, hemorrhage, or vascular malformations all pose an immediate life-threatening risk due to impaired blood flow and very sudden functional loss [13]. Ischemic stroke-induced damage is the most frequent cerebrovascular pathology. Ischemic and hemorrhagic types of stroke affect all components of the neurovascular unit [244]. Activation of brain endothelial cells and surrounding cell types, such as astroglia, pericytes, and microglia leads to proinflammatory cytokine and chemokine production initiating neuroinflammation. Downstream steps in stroke pathology are BBB opening and immune cell entry to the brain, causing further BBB dysfunction and worsening inflammation and edema [245]. Nutraceuticals improve stroke outcomes and inflammation in multiple animal models of the diseases (Table 2).

**Figure 3:**
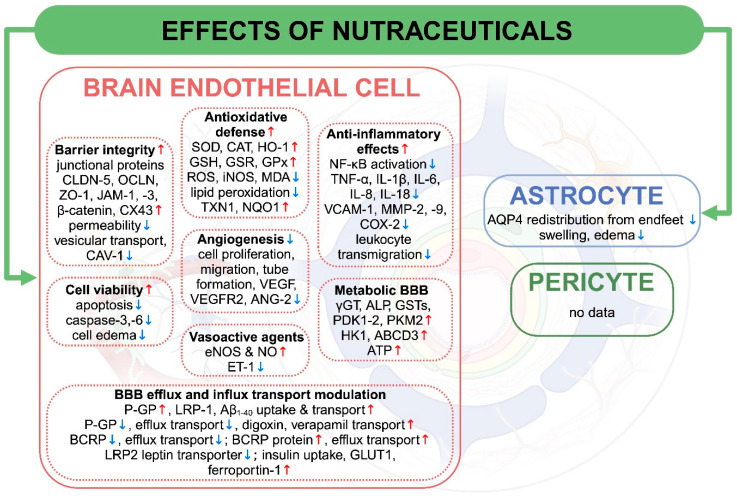
The molecular basis of the protective effects of nutraceuticals on cells of the blood–brain barrier. Elements of brain endothelial cell and astrocyte functions that are involved in BBB damage and rescued by nutraceuticals are shown. ABCD3: ATP-binding cassette sub-family D member 3; ALP: alkaline phosphatase; ANG-2: angiopoietin 2; AQP4: aquaporin-4; ATP: adenosine triphosphate; Aβ_1–40_: amyloid-β_1–40_; BCRP: breast cancer resistance protein; CAT: catalase; CAV-1: caveolin-1; CLDDN-5: claudin-5; COX-2: cyclooxygenase-2; CX43: connexin-43; eNOS: endothelial NOS; ET-1: endothelin 1; GLUT1: glucose transporter 1; GPX: glutathione peroxidase; GSH: glutathione; GSR: glutathione-disulfide reductase; GSTs: glutathione S-transferases; HK1: hexokinase Type I; HO-1: heme oxygenase-1; IL-1β; -6; -8; -18: interleukin-1β; -6; -8; -18; iNOS: inducible nitric oxide synthase; JAM-1; -3: junction associated molecule-1; -3; LRP1; -2: low density lipoprotein receptor-related protein 1; MDA: malondialdehyde; MMP-2; -9: matrix metalloproteinase-2; -9; NF-κB: nuclear factor kappa-light-chain-enhancer of activated B; NO: nitric oxide; NQO1: NAD(P)H quinone oxidoreductase 1 and -2; OCLN: occludin; PDK1; -2: pyruvate dehydrogenase kinase 1; -2; P-gp: P-glycoprotein; PKM2: pyruvate kinase isozyme type 2; ROS: reactive oxygen species; SOD: uperoxide dismutase; TNF-α: tumor necrosis factor-α; TXN1: thioredoxin 1; VCAM-1: vascular cell adhesion molecule-1; VEGF: vascular endothelial growth factor; VEGFR2: vascular endothelial growth factor receptor 2; ZO-1: zonula occludens-1; γGT: gamma-glutamyl transpeptidase; ↑: increase; ↓: decrease. Created in BioRender. Kucsápszky N.; Santa Maria; A. (2025) https://BioRender.com/g20w064 (accessed on 10 February 2025).

Apigenin induced neovascularization and decreased cerebral infarction in a rat middle cerebral artery occlusion (MCAO) model [190]. Tangeretin, quercetin alone and in combination with hydroxysafflor yellow A, curcumin, hesperidin, polydatin, procyanidin B2, pterostilbene, docosahexaenoic acid (DHA), eicosapentaenoic acid (EPA), and ferulic acid decreased brain edema and increased BBB integrity [83,200,203,204,205,208,225,226,227,228,229,231,235,238]. Curcumin in a hypoxia–hypercapnia model counteracted brain edema formation by decreasing the gene and protein expression of aquaporin 4 (AQP4) water channels [201] found in astrocytic endfeet wrapping brain capillaries (Figure 3). In a model of recurrent ischemic attacks, resveratrol decreased brain water content and improved barrier properties, without a change in cerebral blood flow [236]. Rosmarinic acid protected the BBB against MCAO-induced damage in diabetic rats [237]. DHA and EPA, quercetin, hesperidin, polydatin, procyanidin B2, ferulic acid, and vitamin C also elevated the expression of tight junction (TJ) and junctional associated molecules in animal MCAO models [83,205,208,225,226,227,240,241]. Treatment after reperfusion with DHA and EPA, quercetin, ferulic acid, pterostilbene, and vitamin C increased barrier integrity by decreasing the expression of MMP-2 and/or MMP-9 [205,208,229,231,240]. The kaempferol glucoside juglanin showed similar effects after MCAO by the inhibition of the vascular endothelial growth factor (VEGF)/VEGFR pathway [215]. The same observations were made for naringenin and quercetin, which modulated the GSK-3β/β-catenin pathway [224,232], while green tea polyphenols decreased the level of PKCα [207]. Polydatin downregulated the expression of proinflammatory markers and cell surface molecules in a rat brain ischemia–reperfusion injury [226]. In a mouse MCAO model, β-carotene in emulsified or nanocarrier form increased barrier integrity, ZO-1, and occludin levels, and decreased the production of peroxynitrite that lowers mitochondrial membrane potential and enhances cell apoptosis and necrosis [196]. Daidzein restored the ultrastructural changes at the BBB in a rat model of stroke, decreased astrocyte swelling, cytoplasmic vacuolation, edema, and increased blood vessel lumen [202]. The barrier protective effect of malvidin was also mediated by elevation in eNOS and decrease in MMP-9 [223].

The monoterpene borneol (Table 1, Figure 1) has a dual effect on the BBB. On the one hand, borneol and its derivatives are protective in models of cerebral ischemia–reperfusion injury [111,194]. At a wide range of doses (0.5–600 mg/kg body weight), borneol relaxed blood vessels, and decreased blood pressure and cerebrovascular resistance through lowering endothelin-1 and elevating eNOS expression. Borneol restored BBB integrity and decreased edema in the brain by increasing claudin-5 and ZO1 expression, decreasing MMPs and brain endothelial cell surface adhesion molecules intercellular adhesion molecule-1 (ICAM-1) and lymphocyte function-associated antigen-1 (LFA-1) [111,194].

On the other hand, the BBB opening effect of borneol is well documented. Increased entry of the lipophilic efflux pump substrate rhodamine-123 was described in the hippocampus and hypothalamus of healthy rats treated with 100 or 200 mg/kg body weight borneol for one week [193]. This BBB opening effect was explained by the disruption of brain endothelial tight junctions by transmission electron microscopy and the decreased expression of PGP and multidrug resistance protein MRP1 (Table 2). Borneol not only improved the brain entry of a variety of therapeutic molecules, but also enhanced drug delivery across other biological barriers [186]. Several mechanisms can contribute to the drug penetration enhancing effect of borneol. First, due to its lipophilic nature and small molecular weight, it can form micelles in lipid membranes, which will perturb plasma membrane lipid structures and lead to interendothelial tight junction disruption and enhanced cell membrane permeability [186]. Second, it can increase BBB opening by activation of adenosine receptors [246]. Third, it can elevate the entry of lipophilic and amphiphilic drugs and xenobiotics by inhibiting efflux pumps [186,193]. Based on these effects, borneol is investigated as a potential permeability enhancer for better drug delivery [186].

In a general hypoxia model in mice, cerebral edema was reversed by coumaric acid, similarly to dexamethasone. BBB integrity, occludin levels, and brain edema returned to the level of the non-hypoxic animals [199]. Although atherosclerosis affects mainly larger vessels in the brain [13], the BBB is also damaged during hyperlipidemic conditions. Our laboratory described that an ApoB-100 mouse model for hypertriglyceridemia ([247], Bjelik et al., 2006) presented increased BBB permeability, edema and TJ disruption along with gene expression changes [248]. In this mouse model, proinflammatory interleukin-6 (IL-6) levels were also upregulated [249]. In high-fat-diet-induced rat hyperlipidemia, lycopene restored the level of TJ molecule claudin-5, and decreased VEGF, vascular cell adhesion molecule-1 (VCAM-1) and proinflammatory cytokines [222]. In a rat diabetic model induced by high-fat diet and streptozocin, luteolin increased ZO-1, occludin, and GLUT-1 expression [220].

In a subarachnoid hemorrhage model, apigenin decreased inflammation and BBB disruption by elevating ZO-1 and occludin expression [189]. Hemorrhage-induced vasospasm was decreased by lutein [217]. Astaxanthin-derivative adonixanthin and lycopene lowered BBB leakage during subarachnoid hemorrhage [191,221]. Astaxanthin increased barrier integrity and decreased brain edema through the downregulation of MMP-9 expression [192].

#### 3.1.3. Neurodegenerative Diseases

Changes in BBB functions, namely permeability increase and decreased expression of transporters, such as GLUT-1 and P-glycoprotein, already occur during the preclinical, mild cognitive impairment stage of neurodegenerative diseases, such as Alzheimer’s disease. At later stages, severe BBB damage with microbleeds, leukocyte infiltration, and perivascular neurotoxic aggregates are present [12]. In an animal model of memory deficit induced by β-amyloid peptide 25–35 (Aβ_25–35_), luteolin increased barrier integrity by upregulating the expression of junctional associated molecule ZO-1 and TJ proteins occludin and claudin-5. It also decreased astrocyte swelling, and increased cerebral blood flow [219]. In a similar model, quercetin protected BBB integrity and elevated cerebral blood flow [230].

#### 3.1.4. Neuroinflammation, Oxidative Stress, and Other Neurodevelopmental Diseases

During systemic inflammation barrier integrity, signaling pathways, transport for molecules, and immune cell trafficking can change at the BBB, all altering the normal physiological function [250]. Kaempferol ameliorated the neuroinflammation-inducing effects of LPS in mouse models, resulting in better barrier integrity and higher expression of occludin and connexin-43 [213,214]. In a similar model, kaempferol decreased shrinkage of brain endothelial cells, edema of astrocytes, and the area surrounding the capillaries in the striatum [214]. Folic acid increased BBB integrity in a rat sepsis model [239].

Few investigations focused on environmentally harmful agents, such as polychlorinated biphenyl, which can induce oxidative stress in animals, and also affects BBB permeability. Quercetin restored the level of TJ proteins and TJ-associated proteins at the BBB, and thus protected its barrier function [233].

In uremia, caused by renal damage, toxins are released that also harm the BBB. These patients often need imaging using contrast media that also affect BBB functions. EPA and DHA protected the integrity of the BBB by the upregulation of claudin-5 and laminin [206].

Changes in BBB functions were described in neurodevelopmental diseases, such as autism spectrum disorder [251]. Valproic acid induces symptoms in rats mimicking autism related phenomena. Fisetin elevated barrier integrity and claudin-5 expression in a rat model of autism [209] by possibly modulating the WNT/β-catenin pathway [251].

To sum up, the majority of the in vivo studies on disease models that investigated the effects of nutraceuticals on the BBB focused on barrier integrity (Table 2). BBB permeability changes were measured by brain edema formation and Evan’s blue dye leakage to the brain, which reflects to albumin extravasation. Investigations focused on TJ breakdown and molecular mechanisms leading to the damage of intercellular connections. Since MMPs degrade TJ and basal membrane proteins, their expression was also described in several studies. While upregulation of cell surface adhesion molecules ICAM-1 and VCAM-1 were observed, leukocyte transmigration in these studies was not investigated. Astrocyte function through the regulation of AQP4 levels could be a novel target for nutraceuticals [252]. Another knowledge gap is that changes in the function or morphology of brain pericytes, which have a crucial role in the development and maintenance of the BBB [253], were not explored (Figure 2). Brain endothelial surface glycocalyx is an important element of the barrier functions of the BBB, and is damaged in systemic and brain inflammatory diseases [16]. Since most nutraceuticals listed in this review have antioxidant effects, they might also protect against brain endothelial glycocalyx damage. Investigations on brain pericytes and brain endothelial glycocalyx can be a promising line of research for nutraceuticals in the future. At present, animal models are indispensable tools in CNS research, driving progress toward novel treatments and a deeper understanding of brain health and disease. One should consider, however, the challenges related to animal experiments, including ethical considerations to refine, replace, and reduce in vivo models [254], species-specific differences, and the difficulty of fully recapitulating the human disease phenotypes. Emerging research technologies, especially human organs-on-chip models of diseases, may solve these problems by replacing the current in vivo methods [255].

### 3.2. In Vitro Investigations

In line with global efforts to reduce, refine, and replace animal experiments, cell culture models are on the rise, providing crucial understandings of the basic molecular mechanisms of CNS diseases [13]. Many in vitro models of the BBB exist, from simple cell lines to complex primary cell based co-culture models, from rodent and human origin [256]. The barrier tightness and transporter expression of these models should be characterized before they can be considered suitable to establish disease models or to test compound permeability across the BBB [174,175,256]. The state-of-the-art BBB models are based on human stem cells [175,176], but such models have not been used for the investigation of nutraceuticals yet. In this review, only culture models made from brain endothelial cells or cell lines were included (Table 3).

**Table 3:** In vitro studies: effects of nutraceuticals on brain endothelial cells.

Compound	BBB Model	Injury	Effect on Brain Endothelial Cells	Reference
Apigenin	human BMEC	PMA	tube formation ↓, MMP-9 ↓	[257]
	human BMEC	OGD/R	cell viability ↑, cell migration and tube formation ↑, caveolin-1 ↑	[190]
Astaxanthin and derivatives	human HBMEC cell line	—	proliferation ↑, tube formation ↑ cell cycle G0/G1 phase ↓, S phase ↑,	[258]
		OGD	cell viability ↑, LDH release ↓	
	human BMEC	hemoglobin, collagenase	cell viability ↑, ROS ↓, VE-cadherin ↑	[191]
	mouse bEnd.3 cell line	OGDR	cell viability ↑, apoptosis ↓, FD40 permeability ↓, CLDN-5, ZO-1 ↑	[259]
	porcine BMEC	—	APP, ADAM10 ↑, BACE-1 ↓, PGP, ABCA1, LRP-1, Aβ_1–40_ uptake and transport ↑, cholesterol synthesis ↓	[260]
Borneol	rat BMEC, AC co-culture	—	PGP ↓, R123 accumulation ↑, digoxin, verapamil transport ↑	[110]
	mouse bEnd.3 cell line	—	puerarin, tetramethylpyrazine permeability ↑, ZO-1 ↓	[246]
	rat BMEC	OGD	cell viability ↑, apoptosis ↓, CAT ↑, VEGF and VEGFR1 ↑	[193]
Caffeine	mouse BMEC	TNF-α + IFN-γ	VCAM-1 ↓, iNOS ↓	[261]
Capsaicin	mouse cEND cell line	—	TEER ↓, CLDN5 ↓, ZO-1 dislocation	[262]
	human hCMEC/D3 cell line	TNF-α	IL-1β, IL-6 ↓	[263]
Catechin/Epicatechin and derivatives	rat BMEC, AC, PC co-culture	TNF-α + Il-1β	CLDN-5, β-catenin staining ↑, ROS, NO ↓, leptin transporter LRP2 ↓	[264]
	human BMEC	Aβ_1–42_	Aβ_1–42_ fibril formation ↓, ROS ↓	[265]
Chrysin	mouse bEnd.3 cell line	LPS	VCAM-1 ↓, monocyte adhesion ↓	[266]
Cinnamic acid derivatives	human HBMEC-2 cell line	oxidative stress	cell damage ↓, cell viability ↑, mitochondrial transmembrane potential ↑	[267]
Curcumin	bovine BMEC	oxidative stress	cell damage, LDH release ↓	[200]
	rat BMEC	OGD	LDH release ↓, IL-1β ↓	[268]
	porcine BMEC	—	BCRP protein ↓, efflux transport ↓	[68]
Cyanidin metabolite	HBMEC	hypoxia	cell proliferation ↓, cell viability ↓, cyclin D1, CDK2, CDK4 ↓	[269]
Docosahexaenoic acid	rat BMEC, PC, AC co-culture	oligomeric Aβ42	cell viability ↑, ROS production ↓ barrier integrity ↑ SF, albumin permeability ↓, PGP ↑, R123 accumulation ↓	[270]
	BMEC	OGD	apoptosis ↓	[271]
	porcine BMEC	IL-1β	Calcein-AM accumulation ↑	[272]
Fisetin	human BMEC	PMA	tube formation ↓, MMP-9 ↓	[257]
Fucoxanthin	mouse bEnd.3 cell line	mechanical/ stretch	cell viability ↑, apoptosis↓, TEER ↑, γGT ↑, ACSL4 ↓, PINK1, LC3 ↑	[210]
Gallic acid	rat BMEC, AC, PC co-culture	TNF-α + Il-1β	CLDN-5 and β-catenin staining ↑	[264]
Genistein	human BMEC	TNF-α	TNF-α, IL-1β, CCL-1, IL-8, ICAM-1 ↓, leukocyte tr.migration ↓	[273]
	mouse bEnd.3 cell line	Aβ_25–35_	cell viability ↑, ROS, and nitrotyrosine ↓, GSH ↑	[274]
Kaempferol and derivatives	rat RBE4 cell line	—	ecto-ALP ↑, MPP^+^ uptake ↑	[275]
	rat RBE4 cell line	—	ecto-ALP ↑, insulin uptake ↑	[276]
	human BMEC	OGD/R	cell viability ↑, FD permeation ↓, occludin, ZO-1 ↑	[215]
	human BMEC	hypoxia/reoxygenation	cell viability ↑, apoptosis ↓, mitochondrial membrane potential ↑, tube formation ↑, ICAM-1, VCAM-1, IL-1β ↓	[277]
α-Lipoic acid	bEnd.3, rat BMEC	OGD/R	LDH release ↓	[278]
Luteolin	human BMEC	PMA	tube formation ↓, MMP-9 ↓	[257]
	human BMEC, AC co-culture	Aβ1–40	cell viability ↑, TEER ↑, SF and albumin permeability ↓, TNF-α, IL-1β, IL-6, IL-8 release ↓	[279]
Lycopene	mouse bEnd.3 cell line	—	cell viability ↑	[280]
Myricetin	human BMEC	OGD/R	FD70 permeation ↓, TEER ↑, TNF-α, IL-1β and IL-6 ↓, NO and eNOS activity ↑	[281]
	human BMEC	oxidative stress	cell viability ↑	[90]
Naringenin	mouse b.END5 rat RBE4 cell lines	—	concentration and time-dependent cellular uptake	[37]
Naringin	porcine BMEC	—	BCRP protein ↑, efflux transport ↑	[68]
Piceatannol	mouse bEnd.3 cell line	LPS	ICAM-1 and VCAM-1 ↓, iNOS, ROS ↓	[148]
Polydatin	primary rat BMEC	OGD	cell viability ↑, TNF-α, IL-6 ↓ CLDN-5, occludin, ZO-1 ↑	[226]
Procyanidin	rat BMEC	—	PGP activity ↓, efflux transport↓, R123 accumulation ↑	[35]
Pterostilbene	human BMEC	OGD	cell viability ↑, MMP-9 ↓, CLDN-5, ZO-1, VE-cadherin, occludin ↑, F/G actin ↓	[228]
Quercetin and metabolites	rat RBEC1 cell line	—	concentration- and time-dependent cellular accumulation	[282]
	human BMEC	Aβ1–40	cell viability ↑, LDH release ↓ TEER ↑, albumin and SF permeability ↓, ROS ↓, γGT, ALP ↑	[283]
	human BMEC	oxidative stress	cell viability ↑	[90]
	porcine BMEC	—	BCRP protein ↑, efflux transport ↑	[68]
	human BMEC	hypoxia/reoxygenetion	viability ↑, migration, angiogenesis ↑, CLDN-5 and ZO-1 ↑, VCAM-1 ↓, ROS ↓	[284]
	mouse bEnd.3 cell line	*Glaesserella parasuis* infection	Il-6, Il-8, Il-18, TNF-α, MMP-9, ANG-2, ET-1 ↓, ZO-1, occludin, CLDN-5 ↑	[285]
Quercetin-biapigenin nanoparticles	human hCMEC/D3 cell line	oxidative stress	cell viability ↑, TEER ↓	[286]
Quercetin +/− hydroxysafflor yellow A	human hCMEC/D3 cell line	OGD	cell viability ↑, TEER ↑	[235]
Resveratrol	rat BMEC	OGD	cell viability ↑	[236]
	rat BMEC, AC, PC co-culture	TNF-α + Il-1β	albumin permeability ↓, CLDN-5 and β-catenin staining ↑, NO ↓	[264]
Rutin	HBMEC	hypoxia	cell proliferation ↓, cell viability ↓, cyclin D1, CDK2, CDK4 ↑	[269]
Silymarin	human HBEC-5i cell line	AGE	cell migration ↓, tube formation ↓	[287]
Sulforaphane	human hCMEC/D3 cell line	NRF2 gene silencing by siRNA	mitochondrial ABCB10 ↑	[288]
	mouse BMEC	-	GLUT1 ↑, HK1, PDK1, GSK, PKM2, ATP production ↑, NQO1, CAT, GSTs, TXN1, GSR ↑, ABCD3, ABCB6 ↑, ferroportin-1 ↑	[127]
Tangeretin	human HBMEC cell line	OGD	cell viability ↑, ROS and MDA ↓, SOD activity ↑, NO and iNOS ↓	[289]
Theophylline	mouse BMEC	TNF-α + IFN-γ	VCAM-1 ↓, iNOS ↓	[261]
Vitamin E	human HBEC-5i cell line	oxidative stress	cell viability ↑, apoptosis ↓, mitochondrial membrane potential ↑, ROS ↓, GSH ↑, SOD, GPX, CAT ↑, cytosolic HO-1 and NQO1 ↑	[290]

Abbreviations: Aβ: amyloid-β; ABC: ATP binding cassette transporter; AC: astrocyte; ADAM10: A disintegrin and metalloproteinase domain-containing protein 10; AGE: advanced glycation end products; ALP: alkaline phosphatase; APP: amyloid precursor protein; ATP: adenosine triphosphate; BACE-1: β-site of APP cleaving enzyme; beta-secretase; BBB: blood–brain barrier; BCRP: breast cancer resistance protein; BMEC: brain microvascular endothelial cells; calcein-AM: calcein-acetoxymethyl ester; CAT: catalase; CCL: Chemokine (C-C motif) ligand; CDK: cyclin dependent kinase; CLDN-5: claudin-5; ET-1: endothelin-1; FD: FITC-dextran; LDH: lactate dehydrogenase; GLUT1: glucose transporter 1; GSH: glutathione; GSK: glucokinase; glutathione-disulfide reductase; GPX: glutathione peroxidase; GST: glutathione S-transferase; γGT: γ-glutamyl transpeptidase; HK1: hexokinase type 1; HO-1: heme oxygenase 1; IFN-γ: interferon-γ; Il-1β: interleukin-1β; iNOS: inducible nitric oxide synthase; LPS: lipopolysaccharide; MDA: malondialdehyde; MMP: matrix metalloproteinase; MPP^+^: 1-methyl-4-phenylpyridinium; NO: nitric oxide; NQO1: NAD(P)H dehydrogenase (quinone) 1; NRF2: nuclear factor erythroid 2-related factor 2; OGD: oxygen-glucose deprivation; OGD/R: oxygen-glucose deprivation/reoxygenation; PC: pericyte; PDK1: pyruvate dehydrogenase kinase 1; PKM2: pyruvate kinase isozyme type 2; PGP: P-glycoprotein; PMA: phorbol 12-myristate 13-acetate; R123: rhodamine 123; ROS: reactive oxygen species; SF: sodium fluorescein; SOD: superoxide dismutase; TEER: transendothelial electrical resistance; TNF-α: tumor necrosis factor-α; TXN1: thioredoxin 1; VCAM-1: vascular cell adhesion molecule 1; VEGF: vascular endothelial growth factor; VEGFR: vascular endothelial growth factor receptor; ZO-1: zonula occludens-1; ↑: increase; ↓: decrease.

#### 3.2.1. Cell Viability

The effects of nutraceutical compounds on the viability of brain endothelial was studied the most in culture BBB models, but rarely in uninjured, control conditions (Table 3). Nutraceutical compounds at low concentrations are non-toxic, but high, suprapharmacological concentrations of the flavonoids apigenin, genistein, hesperidin, kaempferol, rutin, and quercetin decreased the viability of rat brain endothelial cells measured by the metabolic assay MTT [56]. In contrast, lycopene increased cell viability in mouse bEnd.3 cell line compared to control group [280].

In all of the models, using different modalities to induce cell injury, nutraceuticals exerted protective effects (Table 3). Among the oxidative stress models, cinnamic acid derivatives inhibited H_2_O_2_-induced cell damage, suppressed the decrease in mitochondrial transmembrane potential, and increased cell viability in HBMEC-2 cell line [267]. Myricetin and quercetin, the main components of blackberry polyphenols, were also protective against H_2_O_2_-induced cell toxicity [90]. Quercetin and apigenin nanoparticle formulation counteracted the metabolic activity decrease caused by the oxidant tert-butyl hydroperoxide [286]. Cell injury measured by LDH release and induced by oxidative stress was inhibited by curcumin [200,268].

Fucoxanthin increased cell viability and inhibited apoptosis and ferroptosis after mechanical stretch injury, an in vitro TBI model, in mouse bEnd.3 cell line by modulating ACSL4, PINK1, and LC3genes regulating autophagy [210]. Astaxanthin and its derivatives, adonixanthin and adonirubin, inhibited cell death in a brain hemorrhage human brain endothelial cell model [191].

Luteolin and quercetin elevated brain endothelial cell viability after treatment with Aβ_1–40_ [279,283]. DHA showed similar protection in all the neurovascular cell types treated with an oligomeric Aβ_1–42_ [270]. Genistein also counteracted cell death induced by Aβ_25–35_ in bEnd.3 cells [274].

Stroke is modeled in vitro by oxygen and glucose deprivation (OGD) that can be followed by a reoxygenation period (OGD/R). This was the most investigated pathological BBB culture model for nutraceuticals (Table 3). Astaxanthin, kaempferol and its derivative juglanin, pterostilbene, and quercetin, with or without hydroxysafflor yellow A, resveratrol, apigenin, borneol, DHA, α-lipoic acid, and tangeretin, promoted cell viability after hypoxia or OGD/R in cultured brain endothelial cells [84,190,193,215,228,235,236,258,259,271,278,289]. Polydatin rescued brain endothelial cell viability in an OGD model [226]. Kaempferol and other agents like resveratrol, quercetin, and polydatin in a plant extract increased cell survival and mitochondrial membrane potential in a hypoxia/regeneration model [277]. Vitamin E protected human brain endothelial cells against oxidative-stress-induced cell death and apoptosis [290].

#### 3.2.2. Cell Proliferation, Migration, and Tube Formation

VEGF together with angiopoietins are the most important mediators of angiogenesis in the brain [253]. VEGF, as its name suggests, elevates brain endothelial cell proliferation, but it also enhances BBB permeability; therefore, its levels are reduced after BBB reaches its full maturity, and the barrier tightness is increased. VEGF levels are increased in CNS pathologies, especially after stroke, when angiogenesis can help neuronal survival in the penumbra region [14].

Under healthy conditions, astaxanthin increased brain endothelial cell proliferation and migration in a scratch assay, and also increased tube formation. The resting G0/G1 phase of the cell cycle was decreased, while the more active S phase was increased by astaxanthin [258]. Apigenin, luteolin, and fisetin decreased the tube forming capacity of human brain endothelial cells [257]. Elderberry and elderflower extracts rich in cyanidin metabolite and rutin inhibited cell proliferation by the downregulation of cyclin D1, CDK2, and CDK4 cell cycle promoters in human brain endothelial cells [269].

Advanced glycation end products promote cell migration and tube formation of endothelial cells. Sylimarin decreased this effect through the GSK-3β-dependent inhibition of VEGF release [287]. In contrast, borneol enhanced the level of VEGF, and decreased VEGFR1 expression in rat brain endothelial cells after OGD pushing cell signaling towards angiogenesis [193]. Quercetin enhanced cell migration in a scratch assay, and increased tube formation in a hypoxia/reoxygenation model [284]. In an OGD/R model, apigenin increased caveolin-1 expression, cell migration, and tube formation in human brain endothelial cells via the VEGF pathway [203]. Kaempferol, resveratrol, quercetin, and polydatin, the main components of a plant extract, promoted tube formation and cell layer regeneration in a hypoxia/reoxygenation BBB model [277].

#### 3.2.3. Barrier Integrity

The intercellular connections of brain endothelial cells are composed molecularly of tight and adherens junction proteins linked with junction associated molecules. In CNS and systemic diseases, brain endothelial TJs weaken, causing BBB leakage that worsens disease pathology [12]. On the other hand, opening the TJs between brain endothelial cells can enable paracellular drug delivery [291,292]. Therefore, the question of whether the TJs need to be reinforced or weakened depends on the goal of the study.

Using in vitro BBB models, barrier opening was induced by borneol, which downregulated ZO-1 expression in bEnd.3 cells and enhanced the permeability of other natural compounds [246]. Capsaicin treatment decreased claudin-5 expression and promoted intracellular redistribution of ZO-1 molecules in cEND cells [262]. A quercetin-biapigenin nanoparticle formulation caused a mild, but significant, decrease in the transendothelial electrical resistance of the hCMEC/D3 model [286].

One of the main hallmarks of Alzheimer’s disease pathology is the perivascular accumulation of Aβ peptide aggregates [12]. This pathology can be mimicked by treatment with fibrillary Aβ_1–40_ that damages basic barrier functions, decreases the electrical resistance, and increases the permeability of the in vitro BBB models. These changes were counteracted by luteolin treatment in a human co-culture BBB model [279]. Quercetin restored BBB integrity in a similar system [283]. In a rat co-culture BBB model, impaired barrier tightness measured by decreased resistance and increased permeability induced by oligomeric Aβ_42_ was blocked by the addition of DHA [270].

Myricetin increased barrier integrity measured by resistance elevation and transcellular permeability decrease [281]. Astaxanthin increased barrier integrity and upregulated claudin-5 and ZO-1 expression in b.End3 cells in an OGDR model [259]. Juglanin (kaempferol-3-O-α-L-arabinofuranoside) decreased paracellular permeability and restored occludin and ZO-1 expression in human brain endothelial cells [215]. Interestingly, juglanin significantly increased the expression of these junctional molecules compared to the untreated control group, suggesting a BBB tightening effect of the compound [215]. Metastasis-associated lung adenocarcinoma transcript 1 (MALAT1) is a long, non-coding RNA that protects the BBB after stroke. Polydatin increased the expression of junctional molecules claudin-5, ZO-1 and occludin in an OGD model via MALAT1 upregulation [226]. After 3 and 12 h of OGD injury, the actin structure of human brain endothelial cells was changed, which was reversed concentration-dependently by pterostilbene [228]. Pterostilbene inhibited the redistribution of occludin, claudin-5, and VE-cadherin from the junctions to the cytoplasm. It also increased the expression of occludin, claudin-5, ZO-1, VE-cadherin, and basal membrane component laminin, while MMP-9 was downregulated [228]. Quercetin in combination with hydroxysafflor yellow A increased the resistance of hCMEC/D3 cells in an OGD model [235], while quercetin alone increased claudin-5 and ZO-1 levels in human brain endothelial cells after hypoxia/reoxygenation [284].

Few other in vitro injury models were studied for BBB integrity. Grape phenolic compounds epicatechin, gallic acid, and resveratrol upregulated claudin-5 and β-catenin immunostaining in a triple co-culture model of the BBB treated with proinflammatory cytokines to model neuroinflammation [264]. The astaxanthin derivative adonixanthin increased VE-cadherin levels in human brain endothelial cells treated with hemoglobin and collagenase, pathological factors in brain hemorrhage [191]. To model TBI, b.End3 cells were injured by mechanical stretch. Fucoxanthin elevated resistance and thus barrier integrity in the model [210]. *Glasserella parasuis* bacterial infection causes meningitis in pigs. An infection model with this microbe decreased the junctional expression of ZO-1, occludin, and claudin-5 in bEnd.3 cells, which was reversed by quercetin [285].

#### 3.2.4. Antioxidative and Anti-Inflammatory Effects

The antioxidative effects of nutraceuticals (Table 1) is one of the central elements of their biological actions and protective effects on the CNS and the BBB [182,293]. This can be a direct chemical scavenging of reactive oxygen species (ROS), or an indirect effect by the elevation of the cellular antioxidative defense through regulatory factors [6,14,293]. The latter includes the upregulation of key antioxidant enzymes such as superoxide dismutase (SOD), catalase (CAT), glutathione peroxidase (GPX), glutathione-disulfide reductase (GSR), and downregulation of enzymes producing excess nitric oxide (NO) like the inducible nitric oxide synthase (iNOS/NOS2). While ROSs contribute to inflammation, the main regulators are cytokines and chemokines, which are produced in all cells of the neurovascular unit and are induced by pathological stimuli, such as bacterial lipopolysaccharides (LPS) [294]. As we discuss below, nutraceuticals downregulate the level of proinflammatory cytokines that also contribute to their protective actions on brain endothelial cells (Figure 3).

An example is sulforaphane, which increased the anti-oxidative stress responses and redox signaling in mouse brain endothelial cells by upregulating key enzymes and proteins such as CAT, GSR, NAD(P)H dehydrogenase quinone 1 (NQO1) and thioredoxin 1 (TXN1) [127].

When ischemia damages brain tissue, the mitochondria in brain endothelial cells enhance oxidative stress by decreasing SOD, CAT, and GPX activity, and increasing ROS and malondialdehyde (MDA) levels. Treatment with tangeretin decreased ROS and MDA levels, elevated SOD activity, and decreased iNOS levels [289]. Borneol partly restored the imbalance caused by OGD in brain endothelial cells by increasing CAT expression [193]. ROS levels were decreased, and VCAM-1 upregulation was blocked by quercetin in a hypoxia/reoxygenation model [284]. In a similar setting kaempferol and other agents like resveratrol, quercetin, and polydatin in a plant extract had the same effect, but they also downregulated ICAM-1 expression [277]. Myricetin, polydatin and curcumin decreased proinflammatory cytokine levels in an in vitro OGD/R model [226,268,281].

Proinflammatory cytokine or LPS treatments are often used to model neuroinflammation in an in vitro setting. Caffeine and theophylline inhibited iNOS production and VCAM-1 expression in mouse brain endothelial cells treated with tumor necrosis factor-α (TNF-α) and interferon-γ (IFN-γ) cytokines [261]. Epicatechin counteracted the increase of ROS release in a similar model in a triple co-culture BBB system [264]. In another study, a capsaicin derivative, nitro-capsaicin, inhibited the release of interleukin-1β (IL-1β) and IL-6 and prevented TNF-α-induced inflammation in hCMEC/D3 cells [263]. Cytokine-mediated up-regulation of TNF-α, IL-1β, IL-8, chemokine (C-C motif) ligand 2 (CCL2/MCP-1) and ICAM-1 was reversed by the treatment of brain endothelial cells by genistein. This isoflavone also inhibited leukocyte transmigration across the cultured brain endothelial monolayer [273]. In an LPS-induced injury model, chrysin decreased the expression of VCAM-1, and subsequently inhibited monocyte adhesion to mouse bEnd.3 cells [266]. In a similar model, piceatannol also decreased the presence of VCAM-1 and ICAM-1 on the surface of brain endothelial cells and lowered iNOS levels and ROS [148].

Oxidative stress was also induced in several types of injury models. ROS release was decreased by astaxanthin after treatment with hemoglobin in a human brain endothelial cells modeling hemorrhage in vitro [191]. Vitamin E reduced ROSs and increased the level of glutathione and antioxidant enzymes SOD, GPX, CAT, NQO1, and heme oxygenase 1 (HO-1), as well as the mitochondrial membrane potential in human brain endothelial cells injured by oxidative stress [290].

Fibrillary Aβ_1–40_ increased proinflammatory cytokine levels in a human BBB co-culture model, which could be reversed by luteolin [279]. ROS levels in brain endothelial cells are elevated by fibrillary Aβ_1–40_ which was inhibited by co-treatment with quercetin through SOD activation [283]. DHA also lowered ROS production in neurovascular cell types treated with Aβ oligomers [270]. ROS levels were decreased, while the antioxidant glutathione concentration was increased in brain endothelial cells after treatment with genistein in a Aβ_25–35_ model [274].

#### 3.2.5. Vasoactive Agents

In addition to their specific BBB characteristics, brain endothelial cells, similarly to peripheral vascular endothelium, produce vasoactive agents, like NO, endothelin-1 (ET-1), prostaglandins, and adrenomedullin. This is a neglected area of in vitro BBB research especially in stem cell-derived BBB models, in which endothelial characteristics should be unequivocally demonstrated [295]. NO is a key vasodilator molecule synthesized in brain endothelial cells by the endothelial NOS enzyme (eNOS/NOS3). NO helps maintain BBB functions, and is an important inter- and intracellular signaling molecule [296]. As discussed above, excess NO produced by iNOS contributes to oxidative stress; therefore, NO levels at physiological and in inflammatory conditions are well regulated.

BBB integrity was rescued by myricetin in an in vitro ischemia/reoxygenation model by activating the eNOS/NO pathway in human brain endothelial cells [281]. The elevated NO production in a neuroinflammation model of the BBB in vitro was decreased by grape phenolic compounds epicatechin and resveratrol [264]. In an OGD model, increased levels of NO and iNOS were reduced by tangeretin [289].

Endothelin-1, a vasoconstrictor was upregulated in mouse bEnd.3 cells infected with *G. parasuis*, an in vitro meningitis model, which was reduced by quercetin [285].

Prostaglandins are vasoactive lipid regulators of the cerebral blood flow produced by cyclooxygenase (COX) enzymes. Apigenin, fisetin, and luteolin inhibited increased gene and protein expression of COX-2 induced by phorbol 12-myristate 13-acetate (PMA) in human brain endothelial cells [257]. In OGD models both DHA [271] and polydatin [226] reduced COX-2 levels in brain endothelial cells.

#### 3.2.6. Effects on BBB Efflux and Influx Transport

Efflux transporters provide chemical defense at the level of BBB by actively blocking the entry of neurotoxic compound to the brain [11]. The dysfunction and downregulation of the two most studied efflux transporters at the BBB, PGP, and BCRP is linked to pathological changes in neurodegenerative diseases, like Alzheimer’s and Parkinson’s diseases [12]. Upregulation of efflux transport at the BBB contributes to the barrier functions, while inhibition of efflux pump activity and downregulation of efflux transporter weaken the chemical defense of the brain and increase the brain entry of efflux pump substrate drugs [181]. As we listed in Table 1, many nutraceuticals interact with influx transporters that are crucial to provide nutrients for brain cells [11]. The most important influx transporter group at the BBB is the solute carriers that are indispensable to carry hydrophilic nutrients and nutraceuticals across the BBB.

Citrus flavonoids hesperetin, naringenin, and their metabolites are taken up by mouse and rat brain endothelial lines in a concentration and time-dependent manner [37]. Quercetin also showed elevated accumulation with time and concentration in rat brain endothelial cells [282]. Sulforaphan upregulated GLUT1, the most important influx transporter of glucose at the BBB, in mouse brain endothelial cells [127]. In healthy cultured porcine brain endothelial cells, astaxanthin upregulated the levels of PGP efflux pump, low density lipoprotein receptor-related protein 1 (LRP1), the receptor of apolipoprotein E, and apolipoprotein A1. Astaxanthin also enhanced Aβ_1–40_ uptake and transport [260]. Kaempferol increased the uptake of the hormone insulin and the monoaminergic neurotoxin 1-methyl-4-phenylpyridinium (MPP+) in rat RBE4 cells [275,276].

In contrast to astaxanthin, other compounds decreased efflux pump expression and activity (Table 3). Borneol downregulated the expression of PGP and increased the accumulation of rhodamine 123, an efflux pump ligand, in brain endothelial cells and the transport of digoxin and verapamil across the BBB model [110]. Borneol also elevated the permeability of two CNS drugs, puerarin and tetramethylpyrazine via activation of A_1A_ and A_2A_ adenosine receptors, and enhanced BBB permeability in bEnd.3 cells [246]. In rat brain endothelial cells, procyanidin also lowered PGP pump activity leading to decreased efflux transport of rhodamine 123 [35]. BCRP protein level and the efflux transport activity was decreased by curcumin, quercetin, and naringin in brain endothelial cells [68].

In models of injury, protective effects were seen. DHA (30 µM) increased PGP activity and decreased rhodamine 123 accumulation in primary rat brain endothelial cells treated with Aβ oligomers [270]. In porcine brain endothelial cells, DHA (5 µM) increased the accumulation of calcein-acetoxymethyl ester (calcein-AM), a ligand of efflux pumps PGP and MRP1, but did not change plasma membrane fluidity or the expression of PGP, indicating that it decreased the efflux pump activity by other mechanisms [272]. DHA treatment in the same model inhibited the effect of the proinflammatory cytokine IL-1β, which increased efflux pump activity measured by decreased cellular accumulation of calcein-AM (Table 3). Epicatechin, a phenolic compound, counteracted the increased expression of leptin transporter LRP2 in primary rat brain endothelial cells treated with cytokines to induce inflammation [264].

#### 3.2.7. BBB Enzymes and Metabolism

Brain endothelial cells express many enzymes, including phase 1 and 2 drug metabolizing enzymes that act as local protection and detoxification systems and work in tandem with efflux pumps at the BBB [14,174]. From these enzymes, γ-glutamyl transpeptidase (γ-GT) and tissue alkaline phosphatase (ALP/TNAP) are considered as BBB markers, since they are highly enriched in brain endothelial cells. In line with this, high levels of γ-GT can reflect good brain endothelial cell functions and protects against BBB permeability increase in stroke [297].

In human brain endothelial cells injured by Aβ_1–40_ treatment, quercetin increased the level of BBB marker enzymes γGT and ALP [283]. Fucoxanthin elevated γ-GT levels in the mouse bEnd.3 cell line, helping to restore BBB integrity in a TBI model [210]. Kaempferol increased ecto-ALP activity in rat brain endothelial cells, which was linked to increased insulin and MPP+ uptake [275,276], while sulforaphane upregulated glutathione S-transferases (GSTs) that participate in phase 2 drug metabolism at the BBB [127].

Astaxanthin treatment reduced cholesterol synthesis, decreased the activity of β-secretase (BACE1), and upregulated the non-amyloidogenic ADAM10 metalloproteinase in porcine brain endothelial cells [260]. Astaxanthin thus shifted the metabolism of amyloid precursor protein, and more soluble, and less aggregating peptides were generated.

One of the hallmark of brain endothelial cells is the large number of mitochondria that produce ATP for energy-demanding efflux pumps, enzymes, and ion pumps at the BBB. In pathologies, energy demand increases while ATP levels drop. Sulforaphane increased ATP production in brain endothelial cells by upregulating many important metabolic enzymes and mitochondrial transporters including hexokinase type 1 (HK1), pyruvate dehydrogenase kinase 1 (PDK1), glucokinase (GSK), pyruvate kinase isozyme type 2 (PKM2), ABCD3, ABCB6, and ferroportin-1/SLC40A1 [127].

## 4. BBB Signaling Pathways Regulated by Nutraceuticals

**Figure 4:**
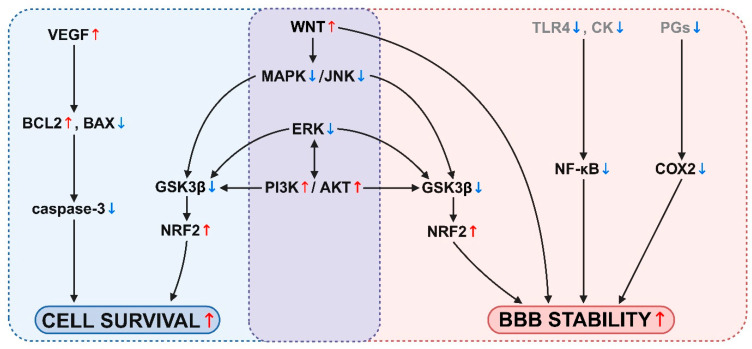
Signaling pathways regulated by nutraceuticals in BBB dysfunction. The simplified drawing shows the most important signaling pathways contributing to the protective effects of nutraceuticals on the BBB. The pathways converge on increased brain endothelial cell survival and BBB stability. BAX: BCL-2-like protein 4; BCL2: B-cell lymphoma 2 protein; CK: cytokines; COX-2: cyclooxygenase-2; ERK: extracellular signal-regulated kinases; GSK-3β: Glycogen synthase kinase-3β; JNK: c-Jun N-terminal kinase; MAPK: mitogen-activated protein kinase; NF-κB: nuclear factor kappa-light-chain-enhancer of activated B; NRF2: nuclear factor erythroid 2-related factor 2; PGs: prostaglandins; PI3K: phosphoinositol 3-kinase; TLR4: toll-like receptor 4; VEGF: vascular endothelial growth factor; ↑: increase; ↓: decrease. Created in BioRender. Kucsápszky N., Santa-Maria, AR. (2025) https://BioRender.com/d25n102 (accessed on 10 February 2025).

The literature on signaling pathways regulated by nutraceuticals is vast, and includes different models and diseases such as tumors or tumor cells, cardiovascular, metabolic, or CNS diseases [298]. From this large knowledge base, we only selected data that prove the regulation of signaling pathways in established BBB preclinical models both in vivo and in vitro (Table 4). As summarized in Figure 4, the most important signaling pathways that mediate the protective effects of nutraceuticals on the BBB converge on increased brain endothelial cell survival and BBB stability.

The VEGF pathway is crucial in brain angiogenesis and BBB development [299]. Brain endothelial VEGF signaling is polarized, and luminal VEGF activates AKT and leads to cytoprotection, while abluminal VEGF increases BBB permeability via p38 [300]. The activation of the VEGF pathway at the BBB was demonstrated by apigenin [190], borneol [193], DHA [271], kaempferol [215,277], and silymarin [287]. VEGF-induced cell survival is mediated by the upregulation of antiapoptotic BCL2 protein, and downregulation of apoptotic BAX protein and caspase-3 and -9 enzymes (Table 4, Figure 4).

The phosphatidylinositol 3-kinase (PI3K)-AKT signaling pathway was the second-most investigated (Table 4) one that leads to the protection of both brain endothelial cells and BBB functions (Figure 4). AKT in brain endothelial cells was activated by cyanidin [301], α-lipoic acid [278], lycopene [280], myricetin [281], quercetin [232] and sulforaphane [127,288]. The inhibition of mitogen-activated protein kinase (MAPK), c-Jun N-terminal kinase (JNK), extracellular signal-regulated kinases (ERK), that crosstalk with the PI3K-AKT pathway, also results in increased brain endothelial cell and BBB protection (Figure 4). The downstream effectors of both PI3K-AKT activation and MAPK-JNK-ERK inhibition are the downregulation/inhibition of glycogen synthase kinase-3β (GSK3β) and the activation of nuclear factor erythroid 2-related factor 2 (NRF2). The translocation of NRF2 to cell nuclei results in the transcription of phase II antioxidant enzymes (Figure 3) that increase the level of antioxidant molecules such as glutathione and decrease the level of ROS [293]. NRF2 activation was observed in BBB models by genistein [274], myricetin [281], quercetin [284], and sulforaphane [127,288]. NRF2 upregulation is also central to the neuroprotective effects of chrysin, naringenin, quercetin and sulforaphane [293], further supporting the link between neuroprotection and BBB protection. Indeed, nutraceuticals that target the NRF2 antioxidant pathway upregulate redox resilience genes, modulate the neurosteroid homeostasis, enhance brain resilience and neuronal adaptive responses in CNS diseases [302].

Despite the importance of the canonical WNT signaling via β-catenin in the development and maintenance of the BBB [253,299], few studies investigated this pathway for natural compounds. WNT activation was observed in BBB models by astaxanthin [191], pterostilbene [228], and quercetin [230] that led to increased BBB stability (Figure 4).

Neuroinflammation is a common feature for CNS diseases and the transcription factor NF-κB mediates the release of proinflammatory cytokines and chemokines in the cells of the neurovascular unit. NF-κB, as a central hub in the inflammatory pathways (Figure 4), was the most investigated signaling molecule in the preclinical BBB models (Table 4). Except for borneol, all of the compounds, namely apigenin [189], capsaicin [263], carvacrol [113], chrysin [266], curcumin [268], gallic acid, resveratrol [264], luteolin [279], piceatannol [148], and quercetin [230] decreased the expression, nuclear translocation, or activity of NF-κB, resulting in BBB protection. Activation of NF-κB also contributes to cell death; therefore, its downregulation/inhibition may also lead to cell survival, but this pathway is not shown in Figure 4.

**Table 4:** BBB signaling pathways regulated by nutraceuticals.

Nutraceutical	BBB Signaling Pathway Interactions	Reference
Apigenin	COX-2 ↓ TLR4, IκB, NF-κB ↓ BECN1 ↓, VEGF, mTOR ↑	[257] [189] [190]
Astaxanthin	caspase-3 ↓, pGSK3β ↓ WNT7A, β-catenin, CCND1 ↑, ERK activation ↓ p75NTR ↓ PPAR-α activation ↑	[258] [191] [259] [260]
Borneol	NF-κB activation ↑ A_1_AR, A_2_AR ↑ BCL-2 ↑, BAX ↓, Ca^2+^ ↓, VEGF ↑, VEGFR1 ↓	[110] [246] [193]
Capsaicin	TRPV1 activity and Ca^2+^ ↑ NF-κB activity, nuclear translocation ↓	[303] [263]
β-Carotene	AKT, FKHR, and ERK1/2 phosphorylation ↓	[196]
Carvacrol	TRPM7 activation ↓ caspase-3 ↓, BAX ↓, BCL-2 ↑, NF-KB ↓	[304] [113]
Catechin/Epicatechin/Epigallocatechin gallate	PKCα ↓	[207] [197]
Chrysin	p38 MAPK and JNK activation ↓, NF-κB p65 translocation ↓	[266]
Curcumin	p38 MAPK and NFκB activation ↓	[268]
Cyanidin	AKT ↑, caspase-3 ↓, ERK1/2 ↓	[301]
Docosahexaenoic acid	ANG2 ↓,VEGF ↑ PGE2, PGI2, COX-2 ↓	[271]
Fisetin	COX-2 ↓	[257]
Fucoxanthin	caspase-3 ↓	[210]
Gallic acid	NF-κB nuclear translocation ↓	[264]
Genistein	NRF2, PI3K ↑	[274]
Hesperidin	FOXO3a nuclear translocation ↓	[85]
Kaempferol and derivatives	VEGF and VEGFR2 ↓ VEGF ↑	[215] [277]
α-Lipoic acid	AKT and mTOR phosphorylation ↑	[278]
Luteolin	COX-2 ↓ NFκ-B activation ↓	[257] [279]
Lycopene	AKT activation ↑, LXR-β ↑	[280]
Myricetin	AKT and NRF2 activation ↑	[281]
Naringenin	p-GSK-3β ↓	[224]
Piceatannol	NF-κB, MAPK, p38, JNK ↓ p-IKKα/β, p-IκBα, p-p65 ↓	[148]
Polydatin	CREB/PGC-1α/PPARγ ↑ COX-2 ↓	[226]
Pterostilbene	c-Met, c-Jun and c-Myc proteins ↑ WNT pathway activation ↑	[228]
Quercetin	KEAP1/NRF2 activation ↑, ATF6/GRP78 ↓ VEGF ↓, PI3K/AKT/ERK activation ↑ WNT ↑, GSK-3β expression ↓ NF-kB p65, RAGE ↓	[284] [285] [232] [230]
Resveratrol	NF-κB nuclear translocation ↓	[264]
Silymarin	VEGF release ↓	[287]
Sulforaphan	NRF2 ↑, AKT phosphorylation ↑ NRF2 ↑	[127,288]
Tangeretin	caspase-3 ↓, JNK activation ↓	[289]
Vitamin E/α-Tocopherol	BAX, caspase-9/caspase-3 ↓, BCL-2 ↑, NRF2 ↑	[290]

Abbreviations**:** A_1_AR: adenosine A1 receptor; A_2_AR: adenosine A2 receptor; ANG2: angiopoietin 2**;** ATF6: activating transcription factor 6**;** BAX: BCL-2-like protein 4; BCL-2: B-cell lymphoma 2 protein; BECN1: Beclin-1; CCND1: Cyclin D1; COX-2: cyclooxygenase-2; CREB: cAMP response element-binding protein; ERK: extracellular signal-regulated kinases; FKHR: forkhead transcription factor; FOXO3a: Forkhead box O3; GRP78: 78 kDa glucose-regulated protein; GSK-3β: Glycogen synthase kinase-3β; JNK: c-Jun N-terminal kinase; KEAP1: Kelch-like ECH-associated protein 1; LXR-β: Liver X receptor-β; MAPK: mitogen-activated protein kinase; mTOR: mammalian target of rapamycin; NF-κB: nuclear factor κ-light-chain-enhancer of activated B; NRF2: nuclear factor erythroid 2-related factor 2; PGC-1α: Peroxisome proliferator-activated receptor gamma co-activator 1α; PGE2: prostaglandin E2; PGI2: prostaglandin I2; PI3K: phosphoinositol 3-kinase; PKCα: Protein kinase Cα; PPARγ: Peroxisome proliferative activated receptor γ; RAGE: receptor for advanced glycation end products; TLR4: Toll-like receptor 4; TRPV1: transient receptor potential cation channel subfamily V member 1; VEGF: vascular endothelial growth factor; VEGFR: vascular endothelial growth factor receptor; WNT7A: Protein Wnt-7a; ↑: increase; ↓: decrease.

COX enzymes produce a complex array of proinflammatory and anti-inflammatory prostanoid and endocannabinoid lipid mediators that are important regulators of neuroinflammation. COX-1 and -2 are important anti-inflammatory pharmaceutical targets for disease modification, and COX inhibitors alone or in combination are examined in clinical trials of neurological diseases [305]. BBB protection by COX-2 inhibition (Figure 4) was observed for apigenin [257], DHA [271], fisetin [257], luteolin [257], and polydatin [226].

Some nutraceuticals can activate or inhibit ion channels at the BBB. The alkaloid capsaicin, an activator of TRPV1, increased intracellular Ca^2+^ levels in cultured brain endothelial cells [303], although no further studies were performed to reveal the functional consequence of the channel activation. The monoterpene carvacrol, a TRPM7 inhibitor, reduced TRMP7 expression in spinal cord vessels, decreased the inflammatory response and stabilized the blood–spinal cord barrier in an injury model [304].

Several nutraceuticals listed in Table 4 belong to the group of phytoestrogens, including curcumin, genistein, kaempferol, resveratrol, and quercetin. These compounds may also exert beneficial effects on the BBB via estrogen receptors, the activation of protective signaling pathways like PI3K-AKT, and inhibition of JNK, ERK, and p38 [202]. BBB protection by phytoestrogens can be mediated by endothelial nitric oxide synthase (eNOS) and the VEGF pathway.

Borneol has a dual, concentration-dependent effect on the BBB. At high concentrations, it increased BBB permeability by activation of NF-κB and adenosin A1A and A2A receptors [110,246]. However, at low concentrations, it also increased BBB stability by elevating BCL-2 and VEGF and reducing BAX and intracellular Ca^2+^ levels [193].

As a conclusion, the most important signaling changes mediating the increased cell survival and BBB stability were the activation of the WNT, PI3K-AKT, and NRF2 pathways, and inhibition of the MAPK, JNK, ERK, and NF-κB pathways (Figure 4). It should be noted that there is crosstalk between many elements of these pathways, and signaling networks at the BBB are very complex and still not fully understood.

## 5. Toxicity and Drug Interactions

This review focused on the protective effects of nutraceuticals, but toxic effects resulting in adverse events and drug interactions were also described for certain nutraceutical supplements [184]. These were reported mainly for dietary supplements containing herbal extracts. Green tea and ginseng extracts were related to acute liver injury, while gingko extracts caused decreased blood coagulation [184]. While most nutraceuticals are considered generally safe, toxicity depends on dose and concentration, and also on individual pharmacogenetics. Caffeine is an example, which, if overdosed, can trigger cardiovascular problems in some individuals [306]. As we described in the cell viability section, the listed compounds were not toxic for brain endothelial cells in the studies, except for very high concentrations of six tested flavonoids [56]. For most of the compounds, toxicity evaluations on different cell types or organ models are missing.

Another potential safety issue is that nutraceuticals by their interaction with drug efflux and influx transporters and drug metabolizing enzymes can cause drug interactions [184]. Most of the listed compounds interact with efflux transporters (Table 1) expressed at the BBB, in the liver, intestines, and kidneys that highly influence the absorption and biodistribution of many prescription drugs such as cyclosporin A, verapamil, digoxin, and many others [11]. The interactions of nutraceuticals with drug efflux pumps may result in higher intestinal absorption, lower excretion, and higher organ penetration of other efflux pump ligand medicines, resulting in their altered pharmacokinetics and pharmacodynamics. These potential detrimental effects of nutraceuticals should be carefully considered in addition to their beneficial or protective properties.

## 6. Conclusions and Future Perspectives

Although the research field of nutraceuticals is promising and provides an almost endless number of molecules to test, the clinical application of nutraceuticals to protect the BBB requires further research. There are several challenges ahead. We should better understand the mechanisms of action of nutraceutical compounds, and optimize their bioavailability and dosing. On the experimental pharmacology level, more critical and systematic reviews would be needed. The preclinical studies reviewed here used single compounds in single disease models. Systematic testing of compound combinations or combinations of nutraceuticals with non-nutraceutical molecules or medicines for synergistic effects and investigations of the same compound on multiple or complex models are missing. Human BBB-on-chip models may provide platforms [295] to solve some of these problems. On the clinical pharmacology level, rigorous and reliable information on the safety and efficacy of nutraceuticals can be obtained from randomized controlled trials [184], which are missing related to the protection of the BBB. This is due to several reasons. The details of BBB dysfunction in diseases, especially clinically, is still not fully understood. Measurements of BBB leakage by dynamic contrast-enhanced MRI or blood biomarkers and BBB dysfunction, like transporter activity or neuroinflammation by PET in patients, are already available [307], but only in few centers. We can expect progress in the adaptation and use of these clinical methods that would accelerate clinical studies on BBB protection in a wide range of diseases. Finally, we cannot expect that nutraceuticals will solve all of the therapeutic problems related to diseases with BBB dysfunction, but they may provide novel active agents or support existing pharmacotherapy. We are optimistic about the future of nutraceuticals as adjunct therapies in combating CNS pathologies and preserving BBB integrity.

## Supplementary Materials

The following information can be downloaded at https://www.mdpi.com/article/10.3390/nu17050766/s1, Table S1: LogP and topological polar surface area (TPSA) values for natural products reported as BBB protective agents. Refs. [308,309,310,311].
