# Supplementary material for: Much More than Nutrients: The Protective Effects of Nutraceuticals on the Blood–Brain Barrier in Diseases
Source: Nutrients. 2025 Feb 21;17(5):766. doi: 10.3390/nu17050766 (PMC11901837; doi:10.3390/nu17050766)
Supplement: Supplementary file 1 [file nutrients-17-00766-s001.zip › nutrients-3467336-supplementary.pdf]

SUPPLEMENTARY MATERIAL TO

Much More than Nutrients: The Protective Effects of Nutraceuticals on the Blood-Brain Barrier in Diseases

Anna E. Kocsis, Nóra Kucsápszky, Ana Raquel Santa-Maria, Attila Hunyadi, Mária A. Deli and Fruzsina R. Walter

Table S1. LogP and topological polar surface area (TPSA) values for natural products reported as BBB protective agents. XlogP3-AA and TPSA values were retrieved from PubChem, Computed by XLogP3 3.0 (PubChem release 2021.10.14) and Cactvs 3.4.8.18 (PubChem release 2021.10.14), respectively. Hydrophilic (H) and lipophilic (L) nature was defined based on negative and positive logP values, respectively; n.a.: logP not available in PubChem, characterized as hydrophilic compounds based on their cationic nature. Characterization of compounds as amphiphilic (A) was based on literature reports.

Nutraceutical	logP	TPSA ($Å^2$)	Solubility	Ref.
**Alkaloids**				
Caffeine	-0.1	58.4	A	[308]
Capsaicin	3.6	58.6	A	[309]
Theophylline	0	69.3	A	[310]
**Anthocyanidines**				
Cyanidin/Cyanidin-3-O-beta-glucoside	n.a.	102	H	
Malvidin/Malvidin-3-*O*-glucoside	n.a.	100/180	H	
**Carotenoids**				
Astaxanthin	10.3	74.6	L	
β-Carotene	13.5	0	L	
Fucoxanthin	8.1.	96.36	L	
Lutein	11	40.5	L	
Lycopene	15.6	0	L	
**Diarylheptanoids**				
Curcumin	3.2	93.1	L	
**Flavonoids**				
Apigenin	1.7	87	L	
Catechin/Epicatechin	0.4	110	L	
Chrysin	2.1	66.8	L	
Daidzein	2.5	66.8	L	
Fisetin	2	107	L	
Genistein	2.7	87	A	[311]

Hesperetin	2.4	96.2	L	
Hesperidin	-1.1	234	H	
Kaempferol	1.9	107	L	
Luteolin	1.4	107	L	
Myricetin	1.2	148	L	
Naringenin	2.4	87	L	
Naringin	-0.5	225	H	
Quercetin	1.5	127	L	
Rutin	-1.3	266	H	
Silybin (complex: Silymarin)	2.4	155	L	
Tangeretin	3	72.5	L	
**Monoterpenes**				
Borneol	2.7	20.2	L	
Carvacrol	3.1	20.2	L	
**Omega-3 fatty acids**				
Docosahexaenoic acid	6.2	37.3	L	
Eicosapentaenoic acid	5.6	37.3	L	
**Organosulfur compounds**				
α-Lipoic acid	1.7	87.9	L	
Sulforaphane	1.4	80.7	L	
**Phenolic acids**				
Caffeic acid	1.2	77.8	L	
Cinnamic acid	2.1	37.3	L	
p-Coumaric acid	1.5	57.5	L	
Ferulic acid	1.5	66.8	L	
Gallic acid	0.7	98	L	
Rosmarinic acid	2.4	145	L	
**Stilbenes**				
Piceatannol	2.9	80.9	L	
Polydatin	1.7	140	L	
Pterostilbene	3.8	38.7	L	
Resveratrol	3.1	60.7	L	
**Vitamins**				
Vitamin C/ Ascorbic acid	-1.6	107	H	
Vitamin B9/ Folic acid	-1.1	209	H	
Vitamin D3/ Cholecalciferol	7.9	20.2	L	
Vitamin E/ α-Tocopherol	10.7	29.5	L	

References

308. Li Y, Luo Z, Li G, Belwal T, Li L, Xu Y, Su B, Lin X. Interference-free Detection of Caffeine in Complex Matrices Using a Nanochannel Electrode Modified with Binary Hydrophilic-Hydrophobic PDMS. ACS Sens. 2021 Apr 23;6(4):1604-1612. doi: 10.1021/acssensors.1c00004.

309. Sharma N, Phan HTT, Yoda T, Shimokawa N, Vestergaard MC, Takagi M. Effects of Capsaicin on Biomimetic Membranes. Biomimetics (Basel). 2019 Feb 13;4(1):17. doi: 10.3390/biomimetics4010017.

310. Konovalova IS, Shishkina SV, Wyshusek M, Patzer M, Reiss GJ. Supramolecular architecture of theophylline polymorphs, monohydrate and co-crystals with iodine: study from the energetic viewpoint. RSC Adv. 2024 Sep 18;14(41):29774-29788. doi: 10.1039/d4ra04368e.

311. Whaley WL, Rummel JD, Kastrapeli N. Interactions of genistein and related isoflavones with lipid micelles. Langmuir. 2006 Aug 15;22(17):7175-84. doi: 10.1021/la0606502.
